# Plasma Thrombospondin-1 in Etiology-Specific Associations with Proteinuria Events in Pediatric Chronic Kidney Disease

**DOI:** 10.3390/children12081101

**Published:** 2025-08-21

**Authors:** Pei-Chen Lu, Wei-Ting Liao, Chien-Ning Hsu, You-Lin Tain, Chia-An Chou

**Affiliations:** 1 Division of Pediatric Nephrology, Kaohsiung Chang Gung Memorial Hospital, Kaohsiung 833, Taiwan; latina@cgmh.org.tw (P.-C.L.); winona0409@cgmh.org.tw (W.-T.L.); tainyl@cgmh.org.tw (Y.-L.T.); 2 Department of Pediatrics, Kaohsiung Municipal Ta-Tung Hospital, Kaohsiung 801, Taiwan; 3 Department of Pharmacy, Kaohsiung Chang Gung Memorial Hospital, Kaohsiung 833, Taiwan; cnhsu@cgmh.org.tw; 4 School of Pharmacy, Kaohsiung Medical University, Kaohsiung 807, Taiwan; 5 College of Medicine, Chang Gung University, Taoyuan 333, Taiwan; 6 Division of Nephrology, Kaohsiung Chang Gung Memorial Hospital, Kaohsiung 833, Taiwan

**Keywords:** children, chronic kidney disease, proteinuria, thrombospondin-1, biomarker

## Abstract

Background: Thrombospondin-1 (TSP-1) is a matricellular protein involved in kidney fibrosis, potentially influencing the progression of proteinuria. However, its potential as a predictive biomarker for proteinuria events in children with chronic kidney disease (CKD), particularly across different etiological subgroups, such as congenital anomalies of the kidney and urinary tract (CAKUT) and non-CAKUT, has not been fully explored. Methods: In this prospective study of 60 children with CKD, we assessed baseline plasma TSP-1 and tracked proteinuria events over one year. Participants were stratified into CAKUT and non-CAKUT groups. Results: In total, 5 of 60 participants had proteinuria events. Plasma TSP-1 was significantly lower in patients with events (21.18 vs. 36.28 μg/mL, *p* = 0.0364). In multivariable analysis, TSP-1 lost significance overall but remained predictive in the non-CAKUT subgroup (AUC = 0.79, *p* = 0.064; OR = 0.93, *p* = 0.028). Conclusions: Plasma TSP-1 may serve as an etiology-specific biomarker for proteinuria events in pediatric CKD, particularly among non-CAKUT patients, and warrants further investigation for personalized risk assessment.

## 1. Introduction

Pediatric chronic kidney disease (CKD) encompasses a highly heterogeneous group of disorders, ranging from congenital structural anomalies to acquired glomerular diseases. Among children with CKD, the most common etiologies are congenital anomalies of the kidney and urinary tract (CAKUT), while non-CAKUT etiologies are often attributable to glomerular disorders such as nephrotic syndrome and proteinuria [1,2]. Regardless of underlying etiology, persistent or recurrent proteinuria is widely recognized as a key driver of CKD progression. Clinically, a sustained urine protein-to-creatinine ratio (UPCR) more than 500 mg/g is a well-established indicator of worsening renal function and increased cardiovascular risk in both adult and pediatric CKD populations [3,4,5]. This highlights the importance of early identification of biomarkers that can predict proteinuria > 500 mg/g, enabling more precise risk stratification and personalized interventions.

Against this background, this study explores the potential role of thrombospondin-1 (TSP-1), a matricellular glycoprotein involved in cell signaling, immune regulation, and extracellular matrix remodeling [6,7,8]. TSP-1 is not kidney-specific; it is primarily stored in the α-granules of platelets and can also be secreted by macrophages, monocytes, and endothelial cells [9]. Its plasma levels are highly responsive to inflammatory stimuli and metabolic disturbances [10,11,12,13]. In diabetes-related research, TSP-1 has been shown to activate transforming growth factor-beta (TGF-β) in tissues, contributing to multi-organ fibrosis [14]. Experimental models further demonstrate that inhibition of the TSP-1/TGF-β interaction can significantly reduce proteinuria and renal fibrosis [15,16]. Additionally, recent clinical data from adult patients with CKD and focal segmental glomerulosclerosis (FSGS) have revealed that elevated plasma TSP-1 levels are significantly correlated with proteinuria severity and CKD progression [17,18]. Importantly, TSP-1 inhibitors are currently under clinical development. Therefore, elucidating the expression pattern and clinical relevance of TSP-1 in pediatric CKD could contribute to the identification of a novel biomarker with both diagnostic and therapeutic implications.

Therefore, the study aim was to evaluate the clinical significance of plasma TSP-1 in children with CKD and assess their association with proteinuria. We hypothesize that plasma TSP-1 levels are positively associated with risk of protein events across etiologically diverse subgroups, supporting its potential role as a predictive biomarker for personalized risk stratification in pediatric CKD.

## 2. Materials and Methods

### 2.1. Study Design and Participants

This was a prospective observational study lasting one year. It adhered to the ethical standards outlined in the 1964 Declaration of Helsinki and its subsequent revisions and received approval from the Institutional Review Board of Chang Gung Medical Foundation, Taoyuan, Taiwan (IRB No. 201801981A3). Before enrollment, written informed consent was signed and obtained from all children as well as their legal guardians.

Participants were individuals aged 3–18 years diagnosed with CKD, recruited from the outpatient clinics of Kaohsiung Chang Gung Memorial Hospital from August 2019 to July 2020. CKD diagnosis and staging followed the guidelines of the National Kidney Foundation’s Kidney Disease Outcomes Quality Initiative (K/DOQI) [19].

The estimated glomerular filtration rate (eGFR) was calculated using the Bedside CKiD equation from the Chronic Kidney Disease in Children study [20]. This equation is applicable to children aged 1–16 years, with the coefficient 0.413 derived from regression modeling in the CKiD study to enable rapid estimation of kidney function. Serum creatinine levels were measured using the enzymatic method in a single laboratory, and height was measured on the same day. The obtained values were entered into the equation to calculate eGFR.

Exclusion criteria were as follows: (a) eGFR less than 15 mL/min/1.73 m^2^; (b) kidney transplantation; (c) ongoing dialysis; (d) congenital heart disease; (e) pregnancy; (f) failure to attend follow-up; and (g) inability to follow the study protocol. Only patients with baseline eGFR > 15 mL/min/1.73 m^2^ who completed full clinical assessments were included in the final analysis.

### 2.2. Clinical Assessments and Specimen Collection

Each participant underwent a comprehensive assessment during a single outpatient visit, including (a) medical history review, physical examination, and office blood pressure (BP) measurement; (b) laboratory data of blood and urine; and (c) 24-h ambulatory BP monitoring (ABPM).

#### 2.2.1. Definition of CAKUT and Non-CAKUT

The causes of CKD were categorized into two groups: CAKUT and non-CAKUT. The CAKUT category included conditions such as renal dysplasia, renal hypoplasia, solitary kidney, reflux-related nephropathy, obstructive nephropathy, and other structural anomalies of the urinary tract. Non-CAKUT encompassed all other etiologies of kidney disease, such as nephrotic syndrome, proteinuria, and hematuria. Proteinuria was defined as a UPCR of at least 200 mg/g, while hematuria was identified by the presence of five or more red blood cells per high-power field in centrifuged urine, confirmed on at least two separate examinations.

#### 2.2.2. BP Monitoring and Criteria for Abnormality

BP assessment was conducted according to the 2017 guidelines of the American Academy of Pediatrics (AAP) [21] and the 2022 recommendations of the American Heart Association (AHA) [22]. All participants underwent office BP measurement, and 24-h ambulatory BP monitoring (ABPM) was performed in individuals aged 6 years or older to ensure reliable data collection, supported by consistent device use and participant cooperation. An abnormal BP profile was considered present if either the office BP or ABPM results met the abnormality criteria, as described by Lee et al. [23].

#### 2.2.3. Plasma TSP-1 Measurement

Plasma TSP-1 levels were measured using a commercial human TSP-1 ELISA kit (Catalog No. CSB-E08763h, CUSABIO, Wuhan, China). This assay employs a quantitative sandwich ELISA technique using microplates pre-coated with anti-TSP-1 antibodies, biotin-labeled detection antibodies, and HRP-conjugated avidin, followed by TMB substrate for colorimetric detection. Samples and standards were assayed in duplicate. Plasma samples were diluted 1:400 in sample diluent prior to testing and incubated at 37 °C, followed by washing and color development. Absorbance was measured at 450 nm. The kit detection range was 2.7–2000 ng/mL, with a sensitivity <2.7 ng/mL. Intra- and inter-assay coefficients of variation were <8% and <10%, respectively. All samples were stored at −80 °C and freeze–thaw cycles were avoided.

#### 2.2.4. Study Outcome Definition

The primary outcome of this study was the occurrence of a proteinuria event, defined as a UPCR > 500 mg/g at any time during the one-year follow-up period, regardless of baseline proteinuria status. Baseline UPCR was measured at the initial clinic visit. Participants were categorized as follows:

Event 1: Participants who experienced a proteinuria event during follow-up (UPCR > 500 mg/g at any point).

Event 0: Participants who did not experience any proteinuria event (UPCR consistently ≤ 500 mg/g) throughout the follow-up.

### 2.3. Statistical Analysis

Data were analyzed using GraphPad Prism (version 10.4.1; GraphPad Software, San Diego, CA, USA) and R software (version 4.5.1; R Foundation for Statistical Computing, Vienna, Austria). Comparisons between groups were conducted with the Mann–Whitney U test for continuous data and either Fisher’s exact test or the Chi-square test for categorical data, as appropriate. Continuous variables are shown as medians with interquartile ranges (IQR), whereas categorical variables are shown as counts and percentages.

To address small-sample bias and zero-event counts in the CAKUT group, multivariable modeling was performed using Firth’s penalized logistic regression via the logistf package in R. Outcomes were expressed as odds ratios (ORs) with 95% profile likelihood confidence intervals. Model adequacy was evaluated using penalized likelihood ratio tests and Tjur’s pseudo R^2^. The potential discriminative ability of plasma TSP-1 was examined through receiver operating characteristic (ROC) curve analysis, with the area under the curve (AUC) and corresponding 95% confidence intervals calculated.

Multicollinearity between independent variables was assessed via variance inflation factors (VIF), with VIF < 5 considered acceptable. All variables included in the model had VIF values close to 1, indicating no substantial collinearity. Pearson’s correlation coefficients were calculated to examine relationships between TSP-1 and clinical parameters in both CAKUT and non-CAKUT groups. Fisher’s z-transformation was used to compare correlation strengths between subgroups.

For the exploratory three-group comparison of TSP-1 and uric acid levels (non-CAKUT with proteinuria events, non-CAKUT without events, and CAKUT without events), unadjusted *p* values were reported for descriptive purposes. All *p* values were two-sided, and statistical significance was defined as *p* < 0.05.

## 3. Results

### 3.1. Clinical and Laboratory Overview

**Figure 1:**
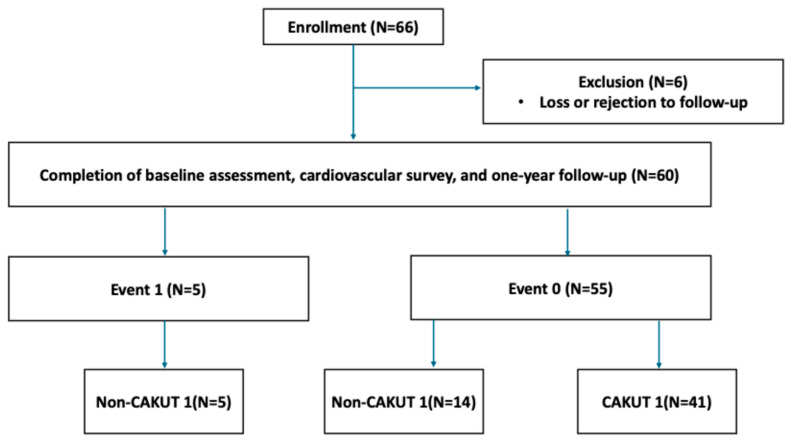
Flowchart of participant enrollment and subgroup classification.

In total, 66 children with CKD were initially enrolled (Figure 1). After excluding six participants due to loss or rejection to follow-up, 60 children completed baseline assessment and cardiovascular survey. Participants were followed for one year and classified based on proteinuria status: Event 1 (proteinuria events, *n* = 5) and Event 0 (no events, *n* = 55).

A total of 60 pediatric CKD participants (median age 10.07 years; 60% male) were included (Table 1). Most patients maintained normal renal function (median eGFR: 101.7 mL/min/1.73 m^2^), with CAKUT being the predominant etiology (68.3%). Baseline anthropometric, biochemical, and metabolic parameters—including BMI, glucose, lipid profile, electrolytes, and hemoglobin—were largely within normal reference ranges. The median UPCR was 89.8 mg/g. Notably, abnormal BP profiles were observed in 63.3% of the cohort. Median plasma TSP-1 level was 34.95 μg/mL (IQR: 26.02–48.19).

### 3.2. Parameters Associated with Proteinuria Events

Among the 60 participants, 5 experienced proteinuria events (Event 1 group), while the remaining 55 did not (Event 0 group). All events occurred exclusively in the non-CAKUT group, which primarily consisted of patients with nephrotic syndrome (n = 3) and isolated proteinuria (n = 2) (Table 2). In contrast, 72.7% of Event 0 group had CAKUT (*p* = 0.0028). Notably, Event 0 group also included patients with similar glomerular pathologies (nine nephrotic syndrome, one proteinuria, two hematuria, and two proteinuria + hematuria).

Event 1 group had significantly higher UPCR levels than Event 0 group did (1386 [789–3143] vs. 84.7 [46.43–240.3] mg/g, *p* = 0.0002) (Table 3). Additionally, Event 1 group had significantly higher serum uric acid levels (9.1 [6.15–12.1] vs. 4.9 [4.2–6.25] mg/dL, *p* = 0.0019) and markedly lower plasma TSP-1 levels (21.18 [8.355–36.08] vs. 36.28 [26.83–48.97] μg/mL, *p* = 0.0364) than Event 0 group did. White blood cell counts were also significantly elevated in the Event 1 group (9.4 [6.95–10.9] vs. 6.5 [5.5–8.1] × 10^3^/μL, *p* = 0.0358). No statistically significant differences were observed between the two groups in terms of age, BMI, eGFR, BP profile, or lipid parameters.

### 3.3. Predictors of Proteinuria Events

To identify potential predictors of proteinuria events and to avoid overfitting due to the small number of outcome events (n = 5), logistic regression analysis was conducted using only the parameters that showed significant differences between the Event 1 and Event 0 groups, including kidney disease etiology (CAKUT vs. non-CAKUT), UPCR, uric acid, WBC, and plasma TSP-1 levels (Table 4). In univariable analysis, higher serum uric acid levels were significantly associated with increased odds of proteinuria events (OR = 2.176, 95% CI: 1.402–4.216, *p* = 0.0002), and lower TSP-1 levels were inversely associated with risk of proteinuria events (OR = 0.9138, 95% CI: 0.8304–0.9832, *p* = 0.0136).

Both kidney disease etiology and serum uric acid levels remained independent predictors of proteinuria events after multivariable adjustment (Table 4). Given the complete absence of CAKUT in the Event 1 group, Firth’s penalized logistic regression was applied to account for data separation. In the adjusted model, non-CAKUT was significantly associated with a higher risk of proteinuria events, consistent with an adjusted OR of 0.0233 for the CAKUT reference group (*p* = 0.0056). Serum uric acid also remained positively associated with events (adjusted OR = 1.8563, 95% CI: 1.11–52.4, *p* = 0.0139). Although TSP-1 exhibited an inverse trend, the association was not statistically significant after adjustment (adjusted OR = 0.9613, *p* = 0.2704).

To investigate why TSP-1 lost significance in the multivariable model, we assessed its potential collinearity with kidney disease etiology and uric acid. Between TSP-1 and uric acid, VIF were low (all <1.05) and the R^2^ was < 0.02, suggesting no meaningful collinearity; thus, the attenuation was unlikely due to uric acid. Although TSP-1 levels did not differ significantly between CAKUT and non-CAKUT groups (*p* = 0.8228; Table S1), all events occurred in the non-CAKUT group. This indicates that the predictive role of TSP-1 may be specific to non-CAKUT (glomerular-based) CKD.

As a sensitivity analysis, we refit the multivariable model to include age and eGFR to assess the potential impact of these standard clinical covariates (Table S2). Neither age nor eGFR reached statistical significance, and the effect estimates for the primary variables remained consistent with the main analysis, supporting the robustness of our findings.

### 3.4. TSP-1 Differentiates Proteinuria Event Risk Among Non-CAKUT Patients

**Figure 2:**
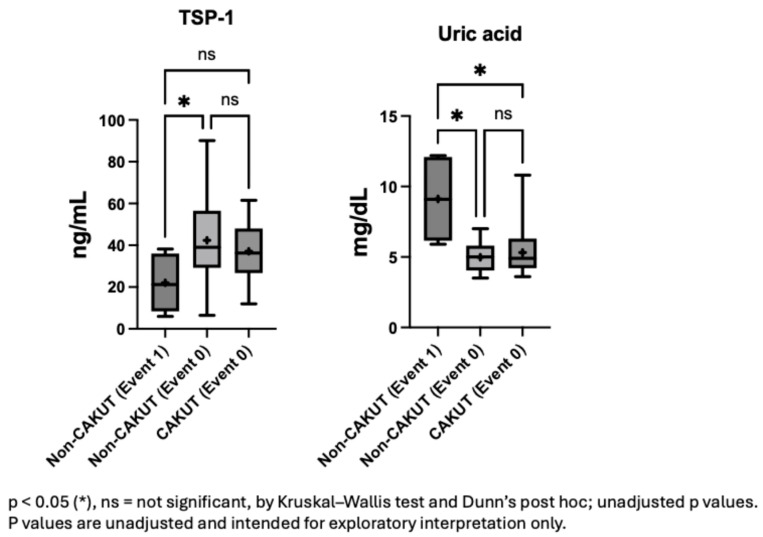
Plasma TSP-1 and uric acid levels stratified by etiology and proteinuria events.

In the non-CAKUT population, compared to those without proteinuria events, individuals with proteinuria events had significantly lower TSP-1 levels (*p* = 0.028; Figure 2). Lower plasma TSP-1 levels may be associated with an increased risk of such events. This suggests that TSP-1 could serve as a reference indicator for clinicians to identify potentially high-risk children among patients with similar glomerular pathologies. In contrast to TSP-1, uric acid levels were higher in the event group, showing significant differences compared to both the non-CAKUT group with similar pathology (*p* = 0.034) and the CAKUT group (*p* = 0.045).

### 3.5. Predictive Value of Plasma TSP-1 for Proteinuria Events in Non-CAKUT Patients

**Figure 3:**
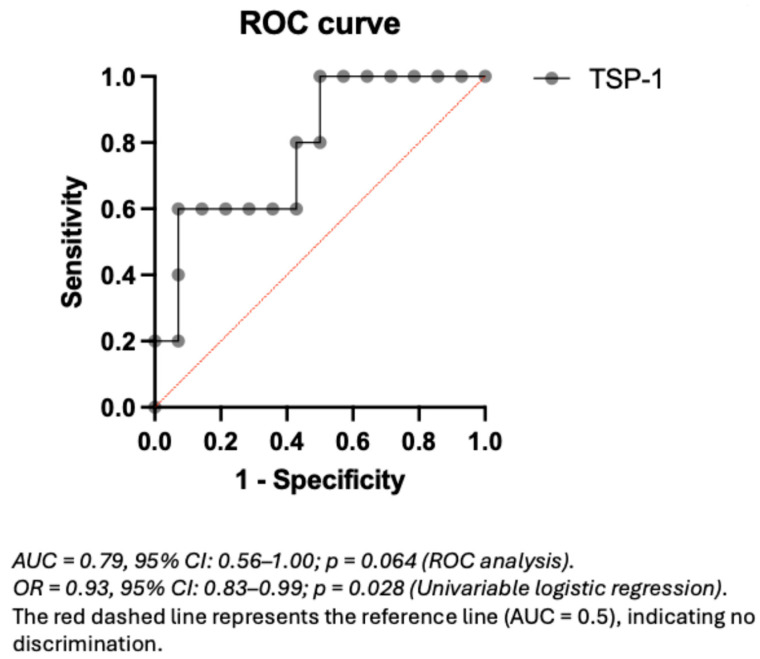
ROC curve of plasma TSP-1 for predicting proteinuria events in non-CAKUT pediatric CKD patients.

To explore the potential predictive value of plasma TSP-1 levels for proteinuria events within the non-CAKUT population, a ROC curve analysis was performed (Figure 3). The AUC was 0.79, which may reflect relatively good discriminative performance. In the non-CAKUT subgroup, a TSP-1 cutoff of 21.18 μg/mL yielded the highest Youden index (0.53), with a sensitivity of 60% and a specificity of 92.9% (Table S3). Within the limitations of the sample size, these findings may point to a relatively high specificity of TSP-1 in relation to proteinuria events in non-CAKUT patients, although further validation in larger cohorts is warranted.

### 3.6. Distinct Clinical Correlates of TSP-1 in CAKUT Versus Non-CAKUT Patients

Given the potential involvement of distinct pathophysiological mechanisms in CAKUT and non-CAKUT patients, subgroup analyses were conducted to explore the correlations between plasma TSP-1 and clinical parameters (Table 5 and Table 6). In the non-CAKUT group (n = 19), TSP-1 showed a statistically significant negative correlation with blood urea nitrogen (BUN) (r = −0.5937, *p* = 0.0074), while no other parameters reached statistical significance. In contrast, in the CAKUT group (n = 41), TSP-1 showed positive correlations with BMI (r = 0.3856, *p* = 0.0128) and hemoglobin levels (r = 0.4637, *p* = 0.0023). These results may reflect different correlation patterns of TSP-1 across etiological subgroups, potentially pointing to differing biological roles of TSP-1 in CAKUT versus non-CAKUT pathology. However, given the limited sample size, these exploratory findings should be interpreted with caution and warrant further validation in larger cohorts.

### 3.7. Fisher’s z-Test Reveals Intergroup Differences in TSP-1–Clinical Correlations

**Figure 4:**
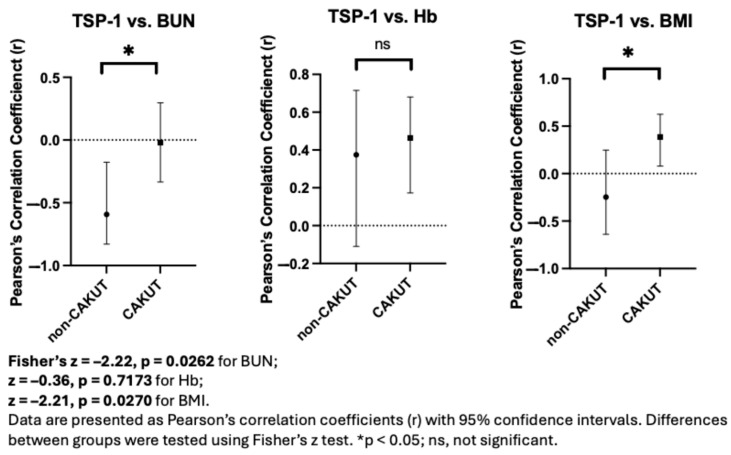
Fisher’s z-test reveals differences in TSP-1 correlations with clinical parameters between CAKUT and non-CAKUT groups.

To further explore whether the strength of association between TSP-1 and clinical parameters varied by CKD etiology, Fisher’s z-transformation was applied to compare Pearson’s correlation coefficients between the CAKUT and non-CAKUT subgroups. Statistically significant intergroup differences were observed for the correlations between TSP-1 and BUN (z = –2.22, *p* = 0.0262) and between TSP-1 and BMI (z = –2.21, *p* = 0.0270) (Figure 4), which may reflect subgroup-specific correlation patterns. No significant difference was found for TSP-1 versus hemoglobin (z = –0.36, *p* = 0.7173). These results raise the possibility that the relationship between TSP-1 and clinical markers could be influenced by underlying disease etiology, highlighting the potential value of stratified analysis in the interpretation of biomarker behavior.

Given that proteinuria events occurred only in the non-CAKUT group, it is possible that TSP-1 is more closely associated with disease activity in non-CAKUT patients, although further investigation in larger cohorts is warranted.

### 3.8. Subgroup Analysis in Non-CAKUT Nephrotic Syndrome Patients

**Figure 5:**
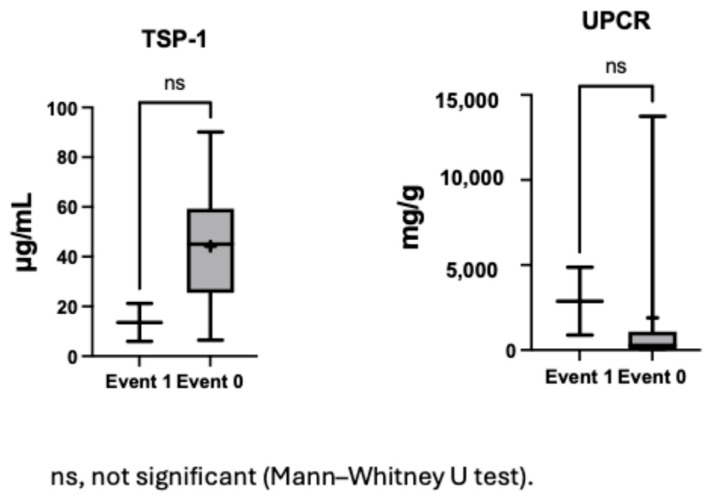
Serum TSP1 and urine protein levels in patients with and without proteinuria events among nephrotic syndrome.

To explore potential associations in a more homogeneous clinical context, a subgroup analysis was performed in patients with nephrotic syndrome without CAKUT. In nephrotic syndrome patients, those who experienced proteinuria events (n = 2) exhibited a trend toward lower serum TSP1 levels and higher urine protein levels compared to those without events (n = 9) (Figure 5). While neither difference reached statistical significance due to limited sample size, these observations point to a possible inverse relationship between TSP1, proteinuria severity, and future proteinuria events, warranting further investigation.

The clinical characteristics of the five patients in the Event 1 group are summarized in Supplementary Table S4. Among them, two patients had relapsing nephrotic syndrome, experiencing four to five episodes per year. One patient with isolated proteinuria exhibited persistent proteinuria. The remaining two patients—one with nephrotic syndrome and the other with isolated proteinuria—showed progressive deterioration in proteinuria and kidney function despite treatment and eventually progressed to end-stage renal disease (ESRD) within one year. Notably, these two patients had the lowest plasma TSP-1 levels (5.96 and 10.75 μg/mL) among all participants.

## 4. Discussion

TSP-1 has long been considered a potentially important regulatory molecule involved in renal fibrosis by activating TGF-β. In adult studies of CKD [17], FSGS [18], and diabetic nephropathy [24], plasma levels of TSP-1 generally increase with disease progression and correlate positively with declining renal function and cardiovascular complications. However, in our study, we observed the opposite trend: in pediatric CKD patients with non-CAKUT (primarily glomerular diseases), those with proteinuria events had significantly lower plasma TSP-1 levels. This suggests that the role of TSP-1 in pediatric CKD may differ fundamentally from its traditionally recognized function in adults.

TSP-1 is a large trimeric glycoprotein (~450 kDa) [25], which is unlikely to pass through the glomerular filtration barrier under normal physiological conditions. The presence of high-molecular-weight proteins—such as IgG (∼150 kDa), larger than albumin (∼67 kDa)—in urine defines non-selective proteinuria and indicates significant structural damage to the glomerular barrier. In such cases, TSP-1 may be excessively lost through urine or deposited in renal tissue, resulting in an artifactual decrease in plasma levels. Previous studies have demonstrated that non-selective proteinuria is strongly associated with increased risks of proteinuria relapse and disease progression [26,27,28]. Therefore, the observed decrease in plasma TSP-1 levels may reflect severe glomerular injury rather than decreased synthesis, further supporting its potential role as a marker of structural renal damage. In our study, plasma TSP-1 levels in the non-CAKUT group were inversely correlated with BUN (r = −0.59), strengthening the link between TSP-1 decline and renal functional deterioration or barrier disruption.

In the CAKUT group, plasma TSP-1 levels were not associated with proteinuria or BUN, but showed positive correlations with BMI and hemoglobin, suggesting that its variation may reflect metabolic, nutritional, or hematologic status rather than active renal injury. While no proteinuria events were observed in this group, proteinuria may still serve as an early indicator of glomerular stress due to adaptive hyperfiltration and remains clinically relevant, given its known association with long-term renal decline. This association is supported by recent longitudinal studies and expert reviews. Walawender et al. reported that proteinuria occurred in 15.2% of patients with multicystic dysplastic kidney and 21% of those with unilateral renal agenesis over long-term follow-up, and that its presence was associated with an increased risk of chronic kidney disease progression [29]. Higher rates were observed in other CAKUT phenotypes, including 18–25% in renal hypodysplasia and up to 45% in posterior urethral valves [29]. In line with these epidemiologic data, Matsell and Catapang emphasized that proteinuria is an established independent predictor of adverse long-term kidney outcomes in CAKUT, particularly in congenital urinary tract obstruction, and has been incorporated—together with baseline and nadir eGFR—into validated clinical risk prediction models for chronic kidney disease [30]. These findings collectively reinforce the rationale for considering proteinuria as an early marker of glomerular stress and a prognostic indicator in children with CAKUT.

In CAKUT, structural abnormalities can lead to reduced nephron numbers (e.g., renal hypoplasia, dysplasia, or solitary kidney) or urinary tract obstruction, which in turn may result in glomerular sclerosis, interstitial fibrosis, and tubular atrophy [31,32]. These fibrotic changes are often associated with increased expression of TGF-β, in which TSP-1 serves as a key activator [32]. While TSP-1 is primarily known for its canonical role in activating latent TGF-β and engaging downstream SMAD-dependent and SMAD-independent pathways (e.g., ERK1/2 MAPK) [33,34,35,36], our findings suggest that plasma TSP-1 levels in CAKUT may not directly correlate with the extent of local renal injury or fibrosis, but instead reflect broader systemic or remodeling processes.

In non-CAKUT renal disease models, TSP-1 may contribute to fibrosis not only through TGF-β activation but also via several TGF-β-independent mechanisms. Its CSVTCG motif within the type 1 repeats binds to CD36, promoting podocyte apoptosis and macrophage TLR4 activation, thereby contributing to inflammation and glomerular injury [37,38]. TSP-1 also antagonizes VEGF and bFGF, leading to capillary rarefaction and fibrosis in unilateral ureter obstruction and aging-related nephropathy models [39,40]. In addition, through its CD47-binding signature domain, TSP-1 suppresses NO-mediated vasodilation and epithelial repair, as demonstrated in ischemia–reperfusion injury models [41,42,43]. In specific disease contexts, the regulation of TSP-1 may be disease-specific. In diabetic nephropathy, TSP-1 expression is upregulated under hyperglycemic conditions via PKC and NF-κB signaling, and further amplified through the AGE–RAGE axis [14]. This contributes to CD36-mediated oxidative and endoplasmic reticulum stress, promoting podocyte apoptosis and proteinuria [14]. However, we observed that in non-CAKUT with proteinuria events, plasma TSP-1 levels were unexpectedly low, suggesting that the plasma TSP-1 does not necessarily correlate with the degree of fibrosis or disease severity. This discrepancy may reflect the dual regulatory nature of TSP-1 in different renal pathologies and could be influenced by disease stage, cellular origin, systemic inflammation, or urinary protein loss. In the nephrotic syndrome patient subgroup, we observed a consistent trend: patients who experienced proteinuria events had lower plasma TSP1 levels and relatively higher urinary protein levels. TSP-1 may be highly expressed locally in kidney tissue but not detectable in circulation, or it may be lost through filtration in the context of massive proteinuria. These findings suggest that plasma TSP-1 may not serve as a universal marker of renal fibrosis across all disease types, and its clinical interpretation should be contextualized within the specific pathological and metabolic environment.

Furthermore, TSP-1 may not be merely a pathogenic factor. Emerging evidence suggests that TSP-1 also exhibits protective properties, including stabilizing vascular integrity, limiting inflammatory spread, inhibiting pathological angiogenesis [44], modulating immune responses, and promoting resolution and repair of inflammation [11,45]. In certain contexts, these functions may contribute to the maintenance of the glomerular filtration barrier. Accordingly, reduced TSP-1 levels may indicate not only glomerular injury but also a loss of its regulatory and protective capacities—potentially increasing the risk of proteinuria relapse. Beyond its role as a predictive biomarker, TSP-1 may also possess therapeutic potential. However, our findings suggest that TSP-1 may exhibit protective functions in certain contexts, indicating a dual biological nature. This highlights the need for therapeutic strategies targeting TSP-1 to be carefully tailored based on disease stage and underlying etiology.

Serum uric acid was a consistent and independent predictor of proteinuria events in both the overall cohort and all subgroup analyses. This finding is supported by multiple publications. In children with steroid-sensitive nephrotic syndrome, higher uric acid levels have been linked to more severe proteinuria and a longer time to remission [46]. Likewise, among adults with primary membranous nephropathy, a serum uric acid concentration above 335 µmol/L is significantly associated with lower remission rates and serves as an important prognostic indicator for proteinuria [47]. The proposed mechanisms include direct injury to glomerular endothelial cells, which increases filtration-barrier permeability and allows proteins to leak into the urine [48]. Uric acid can also amplify oxidative stress and inflammatory responses—activating NADPH oxidase and the NLRP3 inflammasome—to generate large amounts of reactive oxygen species (ROS) and pro-inflammatory cytokines, further damaging glomerular and tubular structures [49]. In addition, it induces fibrosis-related factors such as TGF-β and MCP-1, promoting interstitial fibrosis and tissue remodeling that worsen renal function and proteinuria [48,50]. Importantly, hyperuricemia also leads to glomerular hyperperfusion pressure and vascular constriction, intensifying mechanical stress on the filtration barrier and hastening structural damage [51,52]

In our dataset, we found no significant correlation between uric acid and TSP-1, indicating that they may reflect distinct pathogenic pathways. Therefore, using uric acid together with TSP-1 as complementary biomarkers could enhance risk prediction for proteinuria severity.

To provide a reference framework for interpreting our plasma TSP-1 data, we refer to a pediatric study by Liu et al. [53], which utilized the same ELISA kit (CUSABIO, Wuhan, China). In that study, healthy children had a median plasma TSP-1 level of 100.6 µg/mL (IQR: 58.3–189.5 µg/mL), which appears higher than that observed in our CKD cohort (median: 34.95 µg/mL, IQR: 26.02–48.19 µg/mL). Although direct comparisons across studies should be made with caution due to potential differences in sample handling and participant characteristics, this discrepancy may suggest relatively reduced plasma TSP-1 levels in children with CKD. Such a reduction could potentially be related to altered platelet activity, impaired protein metabolism, or disease-specific regulation of extracellular matrix components. However, further studies including healthy controls within the same population would be necessary to confirm this interpretation.

This study has several limitations. First, it was a single-center study with a relatively small cohort, particularly within the non-CAKUT subgroup (n = 19), limiting the statistical power and generalizability of subgroup analyses. The exploratory findings should be interpreted cautiously and require validation in larger cohorts. Second, plasma TSP-1 was measured only once at baseline. Although the study was prospectively designed, follow-up measurements during relapse and remission, as well as urinary or renal tissue levels, were not collected due to practical constraints. This limits interpretation of temporal biomarker dynamics and mechanistic insights. Third, the absence of a healthy pediatric control group makes it difficult to determine whether the TSP-1 levels observed are pathologically altered. Fourth, proteinuria events occurred only in the non-CAKUT group, precluding evaluation of TSP-1’s predictive value in CAKUT. Lastly, due to the short follow-up duration and the overall preserved renal function at baseline, we were unable to evaluate the predictive value of TSP-1 for long-term eGFR decline. Future longitudinal studies with extended observation periods are warranted.

## 5. Conclusions

In this study, we found that lower plasma TSP-1 levels were significantly associated with proteinuria events in children with non-CAKUT CKD, suggesting its potential role as a significant proteinuria-specific biomarker. The absence of correlation between TSP-1 and traditional markers such as uric acid further supports its ability to reflect a distinct pathological axis. While TSP-1 has primarily been studied in the context of fibrosis and inflammation, our findings highlight its possible dual function—both as a marker of glomerular injury and as a regulator of renal homeostasis. This study has several limitations, including its single-center design, small cohort size, lack of repeated plasma TSP-1 measurements, absence of a healthy control group, and the short follow-up period which precluded assessment of long-term renal decline. Moreover, proteinuria events were observed only in the non-CAKUT group, limiting the evaluation of TSP-1 in CAKUT patients. These factors should be considered when interpreting our findings.

## Figures and Tables

**Table 1:** Clinical and laboratory parameters of the study participants.

Total Participants		n = 60
Baseline Characteristics	Median [IQR]	Unit
Age	10.07 [7.413, 13.9]	years
Male	36 (60%)	n (%)
BMI	17.54 [15.27, 21.16]	kg/m^2^
CAKUT (%)	41 (68.3%)	n (%)
eGFR	101.7 [87.46, 117]	mL/min/1.73 m^2^
UPCR	89.80 [52.20, 279.2]	mg/g
Abnormal BP Profile	38 (63.3%)	n (%)
Fasting Plasma Glucose	89 [84.75, 92.25]	mg/dL
LDL-C	96 [77, 121]	mg/dL
Triglycerides	75 [55, 116]	mg/dL
Uric Acid	5 [4.2, 6.4]	mg/dL
Hemoglobin	13.5 [13, 14.38]	g/dL
WBC	6.85 [5.6, 8.175]	×10^3^/µL
Sodium	139 [138, 141]	mEq/L
Potassium	4.4 [4.125, 4.5]	mEq/L
Calcium	9.9 [9.6, 10.1]	mg/dL
Inorganic Phosphorus	4.8 [4.5, 5.1]	mg/dL
TSP-1	34.95 [26.02, 48.19]	μg/mL

Data are presented as median [IQR] or n (%).

**Table 2:** Diagnostic categories of study participants stratified by CAKUT status and proteinuria events.

Total Participants (n = 60)			
Diagnosis	Non-CAKUT Event 1 (n = 5)	Non-CAKUT Event 0 (n = 14)	CAKUT Event 0 (n = 41)
Nephrotic syndrome	3	9	-
Isolated proteinuria	2	1	-
Isolated hematuria	-	2	-
Proteinuria + hematuria	-	2	-
Renal agenesis, unilateral	-	-	31
Renal hypoplasia	-	-	4
Reflux nephropathy	-	-	1
Hydronephrosis	-	-	2
Renal cyst	-	-	1
Obstructive nephropathy	-	-	2

All values indicate patient counts.

**Table 3:** Comparison of clinical and laboratory parameters between Event 1 and Event 0 groups.

	Event 1 (n = 5)	Event 0 (n = 55)	*p* Value
Age (years)	14 [11.5, 14.47]	9.55 [7.244, 13.53]	0.0772
Male (%)	100%	56.4%	0.0768
BMI (kg/m^2^)	21.27 [16.15, 23]	17.15 [15.27, 21.1]	0.2476
CAKUT (%)	0%	72.7%	0.0028 (**)
eGFR (mL/min/1.73 m^2^)	97.66 [31.84, 123.3]	102.1 [87.5, 116.9]	0.7359
UPCR (mg/g)	1386 [789, 3143]	84.7 [46.43, 240.3]	0.0002 (***)
Abnormal BP Profile (%)	80%	61.8%	0.6432
Fasting Plasma Glucose (mg/dL)	92 [87.75, 96.25]	89 [83.75, 91.25]	0.2388
LDL-C (mg/dL)	133 [90.5, 243]	95.5 [76.75, 114.3]	0.0829
Triglycerides (mg/dL)	213 [57.5, 311.5]	74 [54, 115.3]	0.1215
Uric Acid (mg/dL)	9.1 [6.15, 12.1]	4.9 [4.2, 6.25]	0.0019 (**)
Hb(g/dL)	13.3 [12.95, 14.75]	13.5 [13, 14.4]	0.7600
WBC (×10^3^/µL)	9.4 [6.95, 10.9]	6.5 [5.5, 8.1]	0.0358 (*)
Sodium (mEq/L)	141 [139.5, 141]	139 [138, 141]	0.1438
Potassium (mEq/L)	4.7 [4.15, 4.95]	4.3 [4.1, 4.5]	0.0735
Calcium (mg/dL)	9.7 [8.25, 10.05]	9.9 [9.675, 10.1]	0.2776
Inorganic Phosphorus (mg/dL)	4.9 [4.45, 5.9]	4.75 [4.475, 5.1]	0.4517
TSP-1 (μg/mL)	21.18 [8.355, 36.08]	36.28 [26.83, 48.97]	0.0364 (*)

*p* < 0.05 (*), *p* < 0.01 (**), and *p* < 0.001 (***) by the Mann–Whitney U test and Fisher’s exact test or Chi-square test.

**Table 4:** Univariable and multivariable analysis of predictors for Event 1.

	Univariable				Multivariable		
Variable	OR	95% CI	*p* Value	Note	Adjusted OR	95% CI	*p* Value
Age (years)	1.201	0.9438 to 1.580	0.1367				
eGFR	0.9815	0.9536 to 1.011	0.2074				
UPCR (mg/g)	1.000	0.9998 to 1.001	0.2355				
CAKUT (vs. non-CAKUT)	-	-	0.0028 (**)	Fisher’s exact test	0.0233	1.5 × 10^−10^ to 0.3953	0.0056 (**)
Uric Acid (mg/dL)	2.176	1.402 to 4.216	0.0002 (***)		1.8563	1.11 to 52.4	0.0139 (*)
WBC (×10^3^/µL)	0.6576	0.06111 to 4.829	0.6967				
TSP-1 (μg/mL)	0.9138	0.8304 to 0.9832	0.0136 (*)		0.9613	0.8645 to 1.0247	0.2704

*p* < 0.05 (*), *p* < 0.01 (**), and *p* < 0.001 (***) by univariate and multivariable logistic regression analysis. Multivariable model used Firth penalized logistic regression due to data separation (CAKUT group had 0 events). Multicollinearity diagnostics: VIF for TSP-1 = 1.016, VIF for uric acid = 1.016. No significant collinearity detected (all VIF < 2).

**Table 5:** Correlations of plasma TSP-1 with baseline clinical and laboratory parameters in non-CAKUT patients.

Total Participants			n = 19
Parameter	r	95% CI	*p* Value
Age	−0.2456	−0.6383 to 0.2484	0.3108
eGFR	0.08070	−0.4000 to 0.5265	0.7426
Height	−0.2070	−0.6135 to 0.2862	0.3951
Weight	−0.2651	−0.6505 to 0.2287	0.2726
BMI	−0.2474	−0.6394 to 0.2467	0.3072
Office SBP	−0.1390	−0.5679 to 0.3492	0.5703
Office DBP	−0.2360	−0.6322 to 0.2580	0.3307
LDL-C	0.1187	−0.3815 to 0.5651	0.6390
Triglyceride	−0.2632	−0.6587 to 0.2463	0.2914
Fasting Plasma Glucose	−0.1995	−0.6301 to 0.3249	0.4398
Uric acid	−0.1529	−0.5883 to 0.3513	0.5447
BUN	−0.5937	−0.8299 to −0.1770	0.0074 (**)
Creatinine	−0.1502	−0.5756 to 0.3392	0.5394
UPCR	0.1561	−0.3338 to 0.5796	0.5233
Hb	0.3749	−0.1099 to 0.7156	0.1138
WBC	0.2443	−0.2498 to 0.6374	0.3135
Platelet	0.4088	−0.07022 to 0.7346	0.0823
Sodium	−0.3434	−0.6975 to 0.1455	0.1500
Potassium	−0.2135	−0.6177 to 0.2800	0.3802
Calcium	0.3436	−0.1614 to 0.7060	0.1627
Inorganic Phosphate	−0.4475	−0.7627 to 0.03947	0.0626

*p* < 0.01 (**) by Pearson’s correlation analysis.

**Table 6:** Correlations of plasma TSP-1 with baseline clinical and laboratory parameters in CAKUT patients.

Total Participants			n = 41
Parameter	r	95% CI	*p* Value
Age	0.08981	−0.2329 to 0.3947	0.5766
eGFR	0.1454	−0.1790 to 0.4412	0.3644
Height	0.2070	−0.1168 to 0.4910	0.1942
Weight	0.2833	−0.03607 to 0.5502	0.0727
BMI	0.3856	0.07906 to 0.6255	0.0128 (*)
Office SBP	0.1276	−0.2051 to 0.4338	0.4389
Office DBP	0.1569	−0.1763 to 0.4578	0.3402
LDL-C	0.2505	−0.07126 to 0.5251	0.1141
Triglyceride	0.1702	−0.1543 to 0.4615	0.2875
Fasting Plasma Glucose	0.001179	−0.3151 to 0.3172	0.9942
Uric acid	0.2518	−0.06985 to 0.5261	0.1122
BUN	−0.02100	−0.3349 to 0.2971	0.8963
Creatinine	−0.04026	−0.3519 to 0.2794	0.8026
UPCR	−0.001267	−0.3212 to 0.3189	0.9938
Hb	0.4637	0.1729 to 0.6801	0.0023 (**)
WBC	0.2102	−0.1135 to 0.4935	0.1871
Platelet	0.1415	−0.1828 to 0.4381	0.3774
Sodium	−0.006923	−0.3224 to 0.3099	0.9657
Potassium	0.1387	−0.1856 to 0.4357	0.3872
Calcium	0.2634	−0.05754 to 0.5350	0.0961
Inorganic Phosphate	0.05474	−0.2660 to 0.3646	0.7339

*p* < 0.05 (*), and *p* < 0.01 (**) by Pearson’s correlation analysis.

## Data Availability

The datasets generated and analyzed during this study are not publicly accessible due to concerns regarding patient confidentiality and ethical restrictions. However, they can be obtained from the corresponding author upon reasonable request.

## Supplementary Materials

The following supporting information can be downloaded at: https://www.mdpi.com/article/10.3390/children12081101/s1, Table S1: Plasma TSP-1 Levels in CAKUT vs. Non-CAKUT Groups; Table S2: Multivariable analysis of predictors for Event 1 including age and eGFR. Table S3: Sensitivity, Specificity, and Youden Index Across TSP-1 Thresholds in non-CAKUT; Table S4: Clinical Characteristics of the Five Recurrence Cases.

## Abbreviations

The following abbreviations are used in this manuscript:

ABPM	ambulatory blood pressure monitoring
BMI	body mass index
BP	blood pressure
BUN	blood urea nitrogen
CAKUT	congenital anomalies of the kidney and urinary tract
CKD	chronic kidney disease
eGFR	estimated glomerular filtration rate
ESRD	end stage renal disease
FSGS	focal segmental glomerulosclerosis
HB	hemoglobin
TGF-β	transforming growth factor beta
TSP-1	thrombospondin-1
UPCR	urine protein-to-creatinine ratio
