# Supplementary material for: Plasma Thrombospondin-1 in Etiology-Specific Associations with Proteinuria Events in Pediatric Chronic Kidney Disease
Source: Children (Basel). 2025 Aug 21;12(8):1101. doi: 10.3390/children12081101 (PMC12384188; doi:10.3390/children12081101)
Supplement: Supplementary file 1 [file children-12-01101-s001.zip › children-3745673-supplementary.pdf]

Table S1. Plasma TSP-1 Levels in CAKUT vs. Non-CAKUT Groups.

**Group**	**non-CAKUT**	**CAKUT**	***p* Value**
**TSP-1**	33.98 [25.04–51.19]	36.28 [26.72–48.03]	0.8228

Table S2. Multivariable analysis of predictors for Event 1 including age and eGFR.

**Variable**	**Adjusted OR**	**95%**	**p**
Age (years)	1.14276191	0.81189093-1.60847318	0.44417985
eGFR (mL/min/1.73 m^2)	1.03442099	0.96183275-.11248736	0.36194211
UPCR (mg/g)	0.99975578	0.99907577-1.00043624	0.48167402
CAKUT (vs. non-CAKUT)	0.12082126	0.00666634-2.1897738	0.15278704
Uric Acid (mg/dL)	2.50609098	0.86424271-7.26704657	0.090762
WBC ($\times 10^3$/μL)	1.75633735	0.86592021-3.5623616	0.11852257
TSP-1 (μg/mL)	0.95830514	0.88259998-1.04050392	0.31041916

Adjusted odds ratios (OR) with 95% confidence intervals (CI) are derived from a multivariable Firth's penalized logistic regression model to account for small-sample bias and data separation, given the limited number of outcome events (n = 5). All listed variables were entered simultaneously into the model.

Table S3. Sensitivity, Specificity, and Youden Index Across TSP-1 Thresholds in non-CAKUT.

Threshold (TSP-1)	Sensitivity	Specificity	Youden Index
**5.96**	0.2	1.0	0.20000000000000000
**6.43**	0.2	0.9285714285714290	0.12857142857142900
**10.75**	0.4	0.9285714285714290	0.32857142857142900
**21.18**	0.6	0.9285714285714290	0.5285714285714290
**25.04**	0.6	0.8571428571428570	0.4571428571428570
**25.83**	0.6	0.7857142857142860	0.3857142857142860
**30.35**	0.6	0.7142857142857140	0.3142857142857140
**32.0**	0.6	0.6428571428571430	0.24285714285714300
**33.83**	0.6	0.5714285714285710	0.17142857142857100
**33.98**	0.8	0.5714285714285710	0.37142857142857100
**35.0**	0.8	0.5	0.30000000000000000
**38.18**	1.0	0.5	0.5
**43.16**	1.0	0.42857142857142900	0.4285714285714290
**45.05**	1.0	0.35714285714285700	0.3571428571428570
**51.19**	1.0	0.2857142857142860	0.2857142857142860
**56.15**	1.0	0.21428571428571400	0.2142857142857140
**57.88**	1.0	0.14285714285714300	0.1428571428571430
**60.73**	1.0	0.07142857142857140	0.0714285714285714
**90.09**	1.0	0.0	0.0

Supplementary Table S4. Clinical Characteristics of the Five Recurrence Cases

Case	Age (years)	Gender	Diagnosis	TSP-1 (µg/mL)	UPCR (mg/g)	Treatment	Recurrence Pattern
1	11.57	Male	Nephrotic syndrome	21.18	878	Prednisolone	Relapsing (5 times/year)
2	14.88	Male	Nephrotic syndrome	5.96	4863.6	Prednisolone, cyclosporin, Mycophenolate Sodium	Progressive worsening of proteinuria and kidney function worsening, progressed to ESRD
3	13.99	Male	Nephrotic syndrome	33.98	699.9	Prednisolone, losartan	Relapsing (4 times/year)
4	11.43	Male	Isolated proteinuria	38.18	1421.8	Candesartan	Persist proteinuria
5	14.06	Male	Isolated proteinuria	10.75	1386.1	Candesartan	Progressive worsening of proteinuria and kidney function worsening, progressed to ESRD
